# Supplementary material for: Effects of Inorganic Phosphorus-Solubilizing Bacteria on Rhizosphere Phosphorus Forms and Steroid Saponin Content of Paris polyphylla var. yunnanensis
Source: Biology (Basel). 2025 Sep 17;14(9):1284. doi: 10.3390/biology14091284 (PMC12468006; doi:10.3390/biology14091284)
Supplement: Supplementary file 1 [file biology-14-01284-s001.zip › Supplementary.pdf]

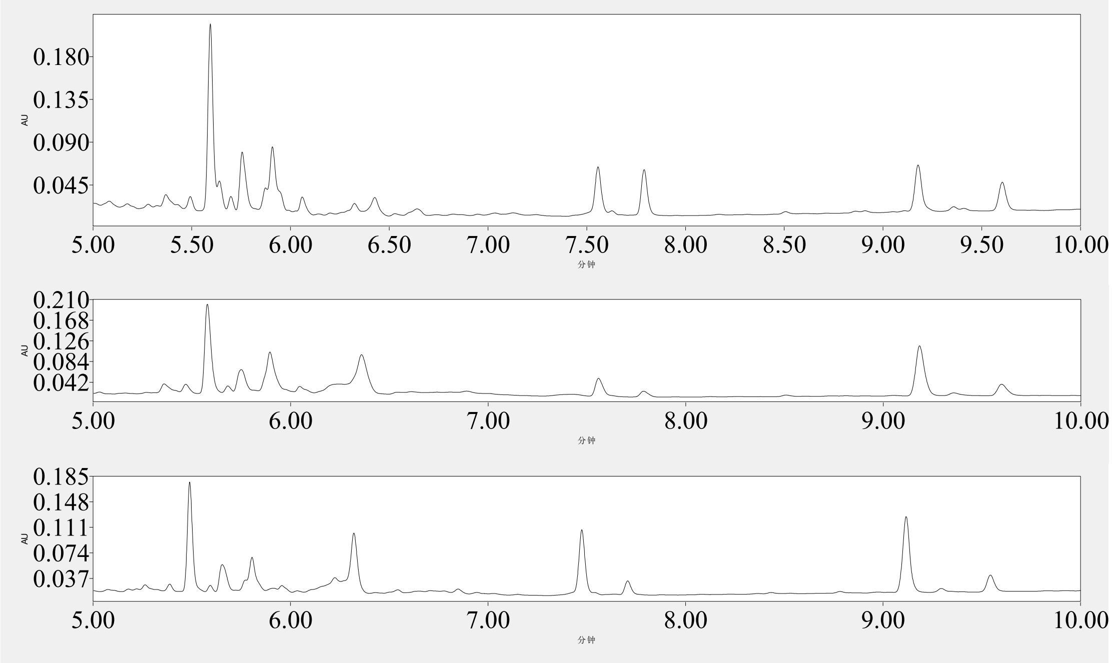

Figure S1. Chromatogram of steroid saponins in sample S1.

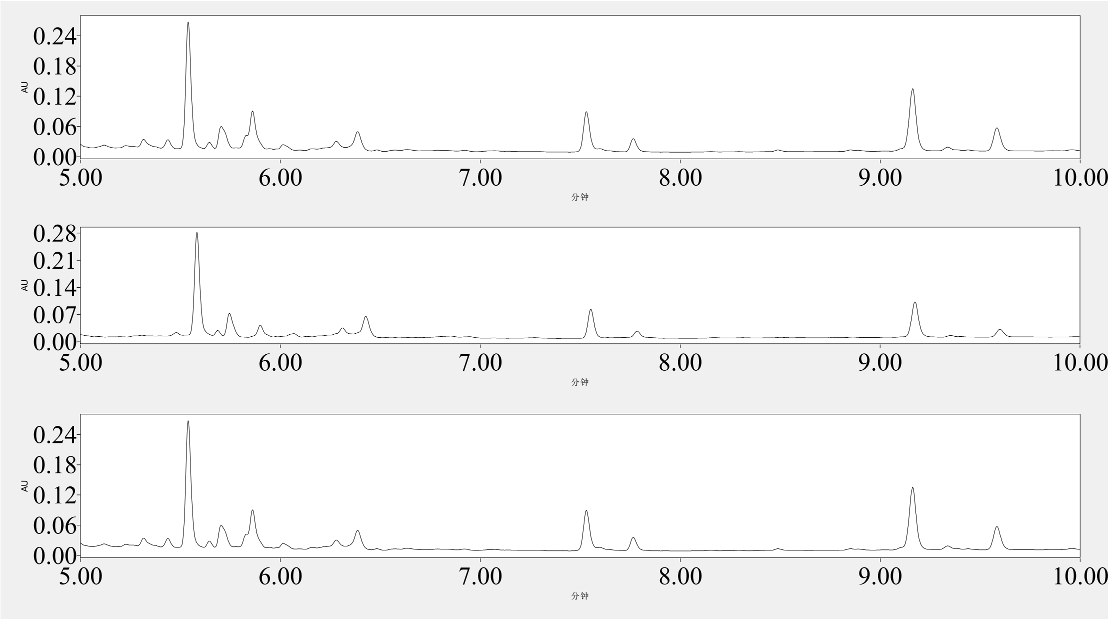

Figure S2. Chromatogram of steroid saponins in sample S2.

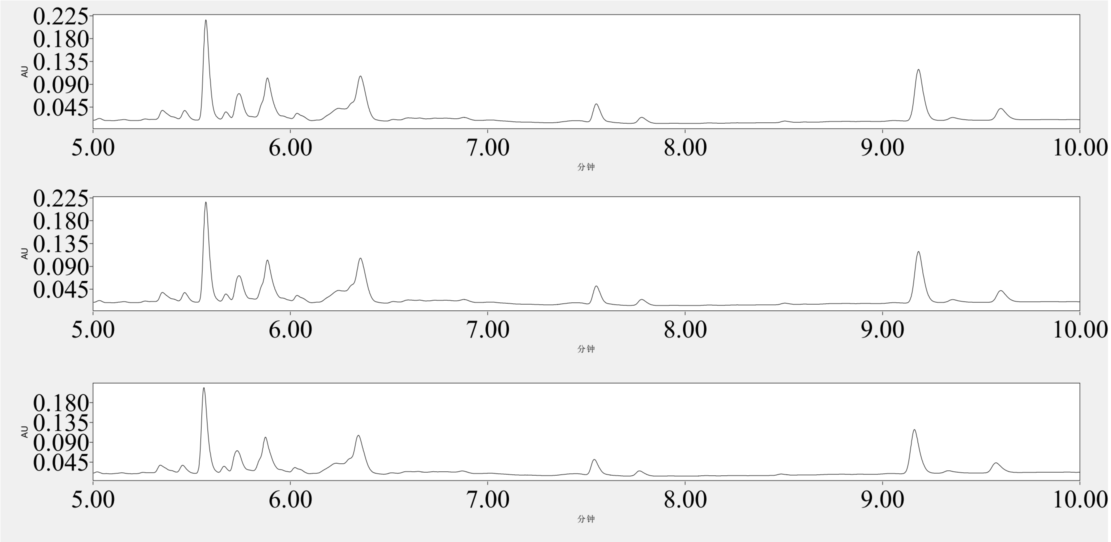

Figure S3. Chromatogram of steroid saponins in sample S3.

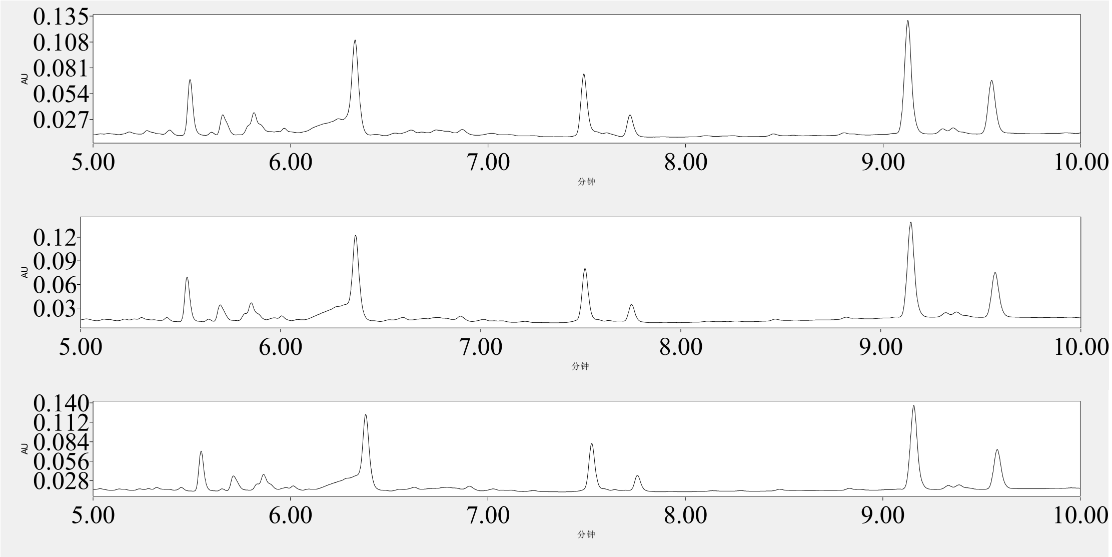

Figure S4. Chromatogram of steroid saponins in sample S4.

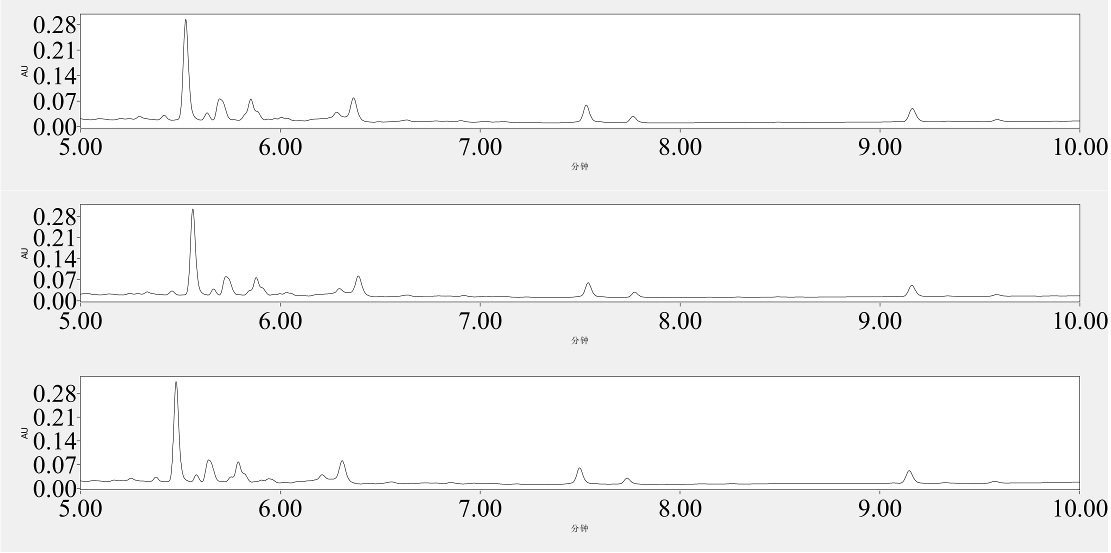

Figure S5. Chromatogram of steroid saponins in sample S5.

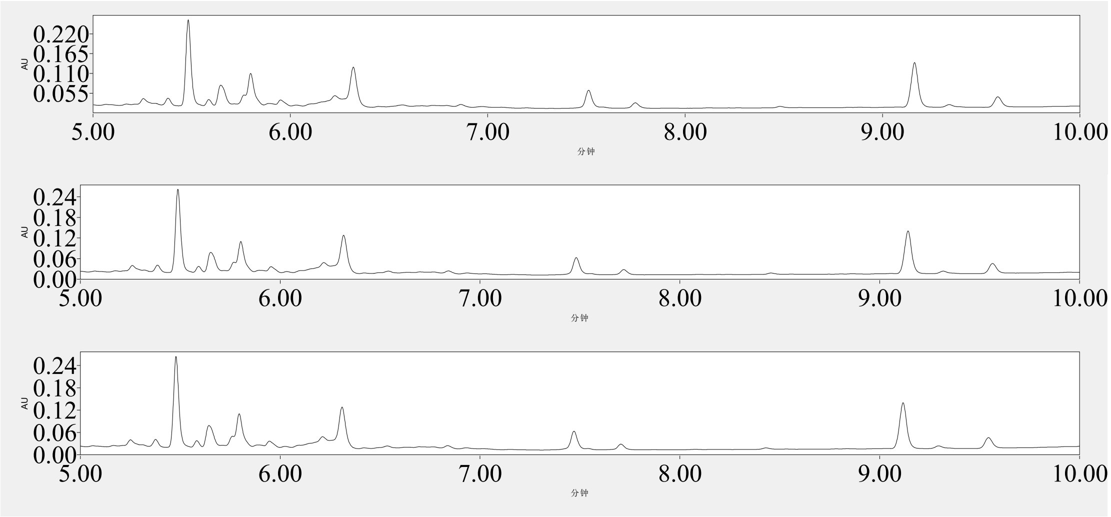

Figure S6. Chromatogram of steroid saponins in sample S6.

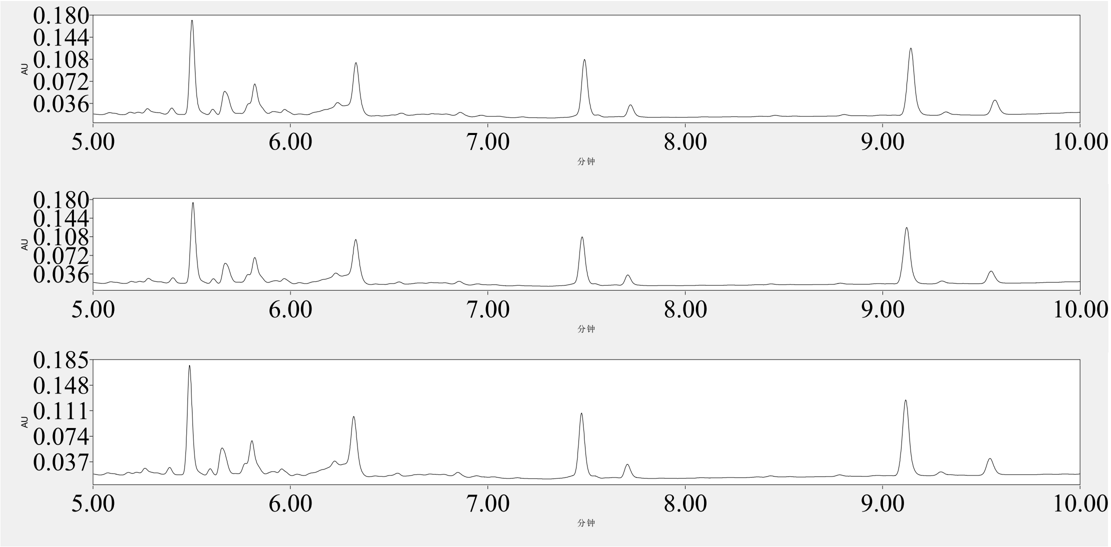

Figure S7. Chromatogram of steroid saponins in sample S7.

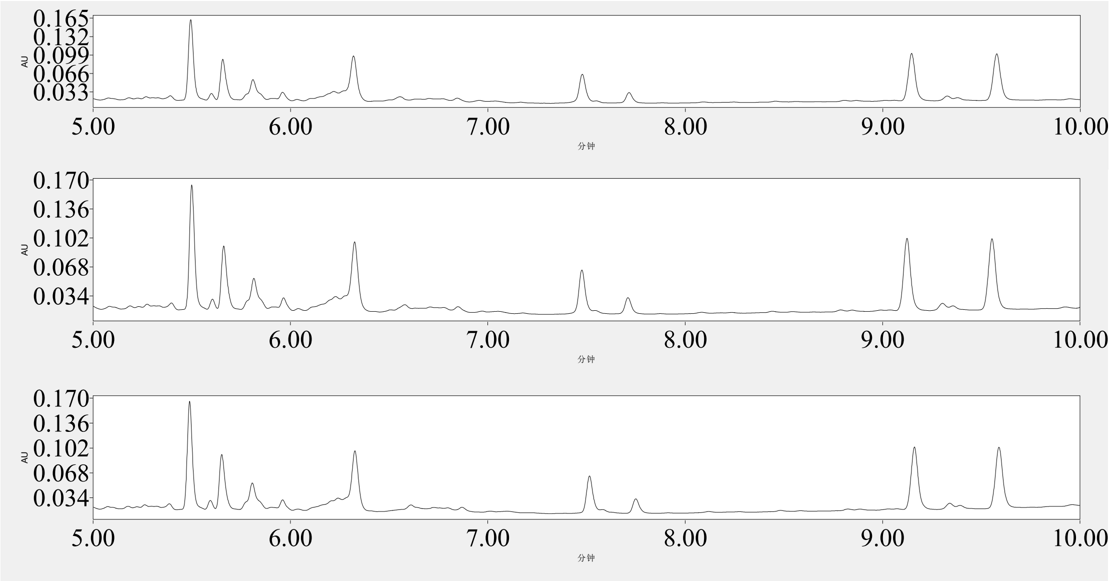

Figure S8. Chromatogram of steroid saponins in sample CK.
